# Identifying Predictive Factors of Recurrence after Radical Resection in Gastric Cancer by RNA Immune-oncology Panel

**DOI:** 10.7150/jca.38536

**Published:** 2020-01-01

**Authors:** Yuehong Cui, Shan Yu, Mengxuan Zhu, Xi Cheng, Yiyi Yu, Zhaoqing Tang, Xuefei Wang, Jun Hou, Yingyong Hou, Dandan Ren, Beibei Mao, Rashid Khalid, Tianshu Liu

**Affiliations:** 1 Department of medical oncology, Zhongshan Hospital, Fudan University, Shanghai, China; 2 Department of general surgery, Zhongshan Hospital, Fudan University, Shanghai, China; 3 Department of pathology, Zhongshan Hospital, Fudan University, Shanghai, China; 4 Genecast Precision Medicine Technology Institute, Beijing, China.

## Abstract

Aiming to identify novel immunotargets for gastric cancer (GC), we retrospectively analyzed the formalin-fixed paraffin embedded (FFPE) samples of gastric cancer tissues from postoperative patients who relapsed or metastasized within (early recurrence, n=25) or after two years (late recurrence, n=23). RNA immune-oncology panel (RIOP) including 398 immune-related genes was used to detect the RNA expression level. Disease free survival (DFS) time in early and late recurrent group was 7.52±0.72 and 28.49±0.81 months, respectively. 18 genes were significantly different between the early and late recurrent groups, and the expression of ITK, EBI3, CX3CL1, MYC, EOMES, CA4, TAGAP, MMP2, HAVCR2, FCGR1 and SNAI2 were verified to be associated with the DFS time. We also found that 18 genes were differentially expressed in diffusal type and non-diffusal type of GC. Leukocyte-inhibition, Leukocyte-migration, and Lymphocyte-infiltrate signal/functional pathways were activated in diffusal type of GC by cluster analysis. Our data uncovered the gene set consisted of ITK, EBI3, and CX3CL1 as a potential tool for prediction of early recurrence or poor prognosis in GC, which could be used as novel immunotargets and prognostic markers for the management of GC.

## Introduction

Gastric cancer (GC) is one of the most lethal and aggressive kind of cancers, being the third cause of cancer-related death worldwide. Most of the cancer-related mortality is caused by metastases formed by disseminated primary tumor cells at distant sites. Even with radical gastrectomy and the latest generation of molecular chemotherapeutics, the numbers of recurrence and mortality remains high. Although these treatments can control many primary tumors effectively, but they offer little in terms of survival benefits in curbing the metastatic spread of cancer cells due to its heterogeneous nature and the ability to evade cell death^1^ and to escape immune system surveillance^2^. Immunotherapy for gastric cancer is one of the emerging therapeutic options, however, it is still in the early phase and needs to be expedited. The clinical benefit and improved survival observed in GC patients treated with immunotherapeutic strategies and their combination with conventional therapies highlighted the importance of the immune microenvironment surrounding the tumor.

There is significant interplay and exchange of communication between tumor cells and the tumor microenvironment (TME) through paracrine signals 3. The immune suppressive TME of GC is composed of many different types of cells, such as tumor- associated macrophages (TAMs), tumor-infiltrating lymphocytes (TILs), cancer-associated fibroblasts (CAFs), and endothelial cells (ECs)^4^. These cells within TME interact and influence each other's functions through production and secretion of various growth factors (GFs), cytokines and chemokines, which are considered to be key orchestrators in the process of proliferation^5^-^6^, inflammation, angiogenesis^7^, and cancer progression^8^-^9^. Interactions between tumor cells and TME protect metastatic cancer cells by diminishing the T-cells functions and the effectiveness of immunotherapy, resulting in the decline of therapeutic outcomes in patients^10^.

The intensive interaction between immune-suppressive TME and the tumor cells plays a key role in the tumor initiation and progression. However, there are various immune related factors, and their mechanism is still not clear, which needs to be further elucidated in order to identify potential prognostic immune markers and therapeutics for the treatment of recurrent GC. Here we evaluated the immunological factors using RNA Immune-oncology panel comprising of 398 immune system relevant genes to compare the difference between early and late recurrence of GC patients after radical resection. We also analyzed the gene expression in diffusal type and non-diffusal type of GC, because the Lauren type, which includes intestinal type, mixed type and diffusal type, is definitely associated with the prognosis. The aim of this study is to find the potential key genes and novel immunotargets that are associated with poor prognosis and recurrence in GC.

## Patients and Methods

### Patients' characteristic

Patients' characteristics were listed in Table 1. We retrospectively collected the clinical data of patients who received radically gastric cancer resection in our hospital from January to December in 2015. All of the patients were stage III according to the AJCC/TNM staging system (7^th^ ed., 2010). The Ethics Committee of Zhongshan Hospital Affiliated to Fudan University have approved this study, and the written informed consent was obtained from each patient before sample collection. We screened total 48 samples of GC patients, which were qualified as for the quality control of RNA extraction. These samples were divided into two groups: early recurrent group (<2 years after surgery) and late recurrent group (≥2 years after surgery)^11^. 25 patients developed recurrence within two years after curative resection. As the control group, 23 patients didn't relapse even after two years of surgery. According to the Lauren type of GC, we considered intestinal type (n=9) and mixed type (n=21) to be non-diffusal type of GC, otherwise they were diffusal type (n=18). The median age of the patients was 60.0 years.

### RNA Immune-oncology panel

An amplicon-based next-generation sequencing (NGS) RNA Immune-oncology panel (RIOP, Beijing Genecast Biological Company) was used to detect the expression of 398 genes at RNA level from FFPE tumor specimens. This research-used only assay was designed to provide a comprehensive characterization for tumor immune microenvironment as which could guide therapeutic decision on patients. The 398 genes (seen in Supplementary Table 1) were characterized into 36 gene functional groups, namely, innate immune response, chemokine signaling, proliferation, apoptosis, antigen presentation, antigen processing, tumor antigen, T-cell regulation, TCR coexpression, dendritic cell, mononuclear-macrophage system, checkpoint pathway, drug target, tumor marker, PD-1 signaling, cytokine signaling, interferon signaling, and so on (Seen in Table 1). Previous unpublished internal validation report has shown that the amplicon-based panel has a higher sensitivity than whole transcriptome sequencing, especially in relatively lowly expressed genes, which is an important requirement for measuring differential expression of immune-related genes.

### The process of RNA extraction for RNA Immune-oncology panel sequencing

To achieve high-quality RNA samples, ultrasonication-based protocol was used for RNA extraction, which included the following steps: (1) Release of DNA, RNA, and protein from cells by ultrasonic crushing; (2) Protein digestion by proteinase K at 56℃ for 15 minutes, then reversal of the crosslink of nucleotide and protein at 80℃ for 1 hour; (3) Digestion the supernant by DNase I; (4) Filtration through RNA combing column together with Buffer B1 and 100% ethanol; (5) After rinsing several times, dissolution of RNA into the elution buffer; (6) Measurement of RNA concentration, and evaluation of RNA quality.

### Reverse transcription and the library construction

(1) The cDNA synthesis from RNA by reverse transcription was performed according to the manufacturer's instruction; (2) Amplifying the targeting fragment by Oncomine Immune-oncology Panel; (3) Partially digesting the amplicons by FuPa Reagent; (4) Ligating the specific connector of amplicons, then purifying and amplifying its product; (5) Construction the library after two cycles of PCR; (6) Measuring the concentration of the cDNA library, and quality control.

### Sequencing

**Figure 1:**
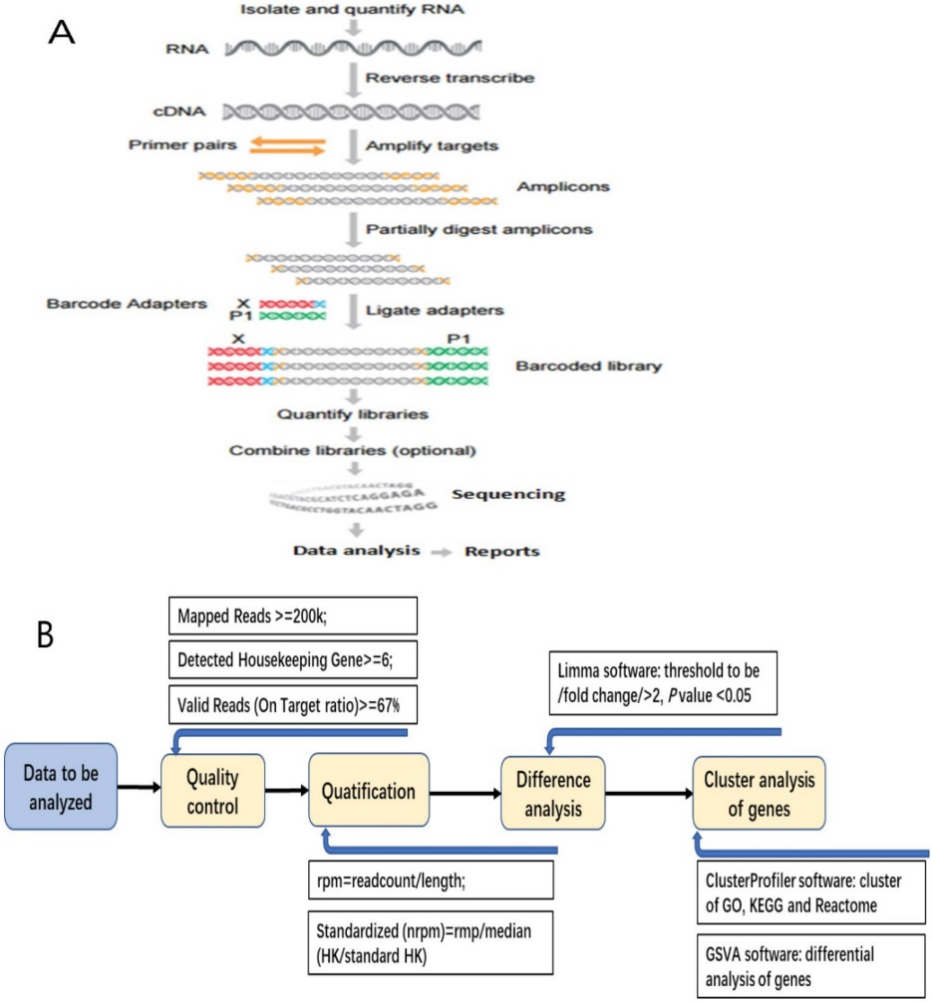
The detecting process of RNA Immune-oncology panel. A: The flow chart of RNA Immune-oncology panel sequencing. B: The flow chart of bioinformatic analysis for the data of gene expression.

After construction of the qualified cDNA library, pooling the data according to the distribution of fragments and the eligible chips, then sequencing was performed on the platform of Ion Torrent S5 (Figure 1A).

### Data analysis

Median DFS was calculated by Independent-Sampled t test using SPSS 17.0 software (IBM, USA). After obtaining the data of RIOP sequencing, quantifying and standardization of the gene expression level, we analyzed the data as follows: (1) Getting the value for each sample from the detected housekeeping gene expression level (rpm) divided by the standard housekeeping gene expression level; (2) Normalized value was obtained from the median of every sample's value; (3) Getting normalized rpm (nrpm) from each sample's rpm divided by normalized value; (4) Analyzing the difference of Log2(nrpm+1) by LIMMA software, and the thresholds of differential screening were: |fold change|≥2, *P* value < 0.05. (5) The cluster analysis for the differential expressive genes was done by using the cluster Profiler Software, including GO, Biological Process, Cellular Component, KEGG, and Reactome (Figure 1B).

## Results

### The survival data between recurrence within or after two years of surgery

There were 35 male and 13 female patients. The median DFS in early and late recurrent groups were 7.52±0.72 and 28.49±0.81 months respectively (*P*=0.000). As for Lauren type, the median DFS time were 21.26±4.39 months in intestinal type, 19.49±2.51 months in mixed type, and 13.48±2.12 months in diffusal type of GC (*P* =0.086). However, if the patients were divided into non-diffusal type and diffusal type groups, the median DFS were 20.02±2.15 and 13.48±2.12 months, respectively (*P*=0.04).

### The difference of gene expression detected by RIOP between early and late recurrent groups

**Figure 2:**
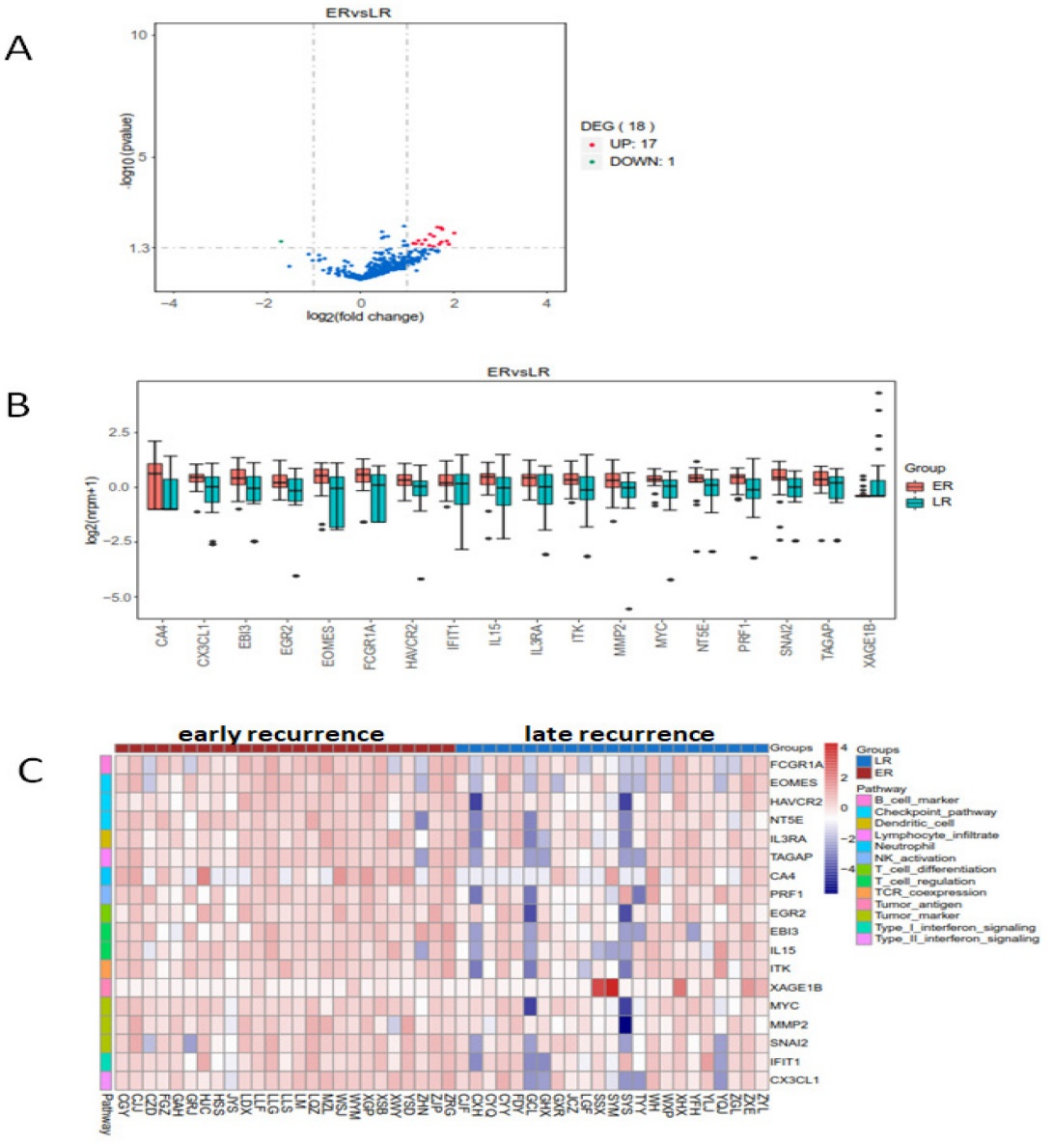
The enrichment analysis of differential expressed genes in early and late recurrent groups. There were seventeen higher and one lower expressed genes in early recurrent group than that in late recurrent group. A: The volcano plot. B: The box plot. C: The heatmap of cluster analysis.

The heatmap of the 398 genes by cluster analysis was generated from log2(nrpm+1) calculation. We compared the genes' expression in early and late recurrent groups by using LIMMA software (moderate t-statistics test, screening condition: |Fold change| ≥2, P value <0.05). The expression of eighteen genes were found differently between early and late recurrent groups. FCGR1A (Fc fragment of IgG receptor 1a), EOMES (eomesodermin), HAVCR (hepatitis A virus cellular receptor), NT5E (5'-nucleotidase ecto), TAGAP (T cell activation RhoGTPase activating protein), CA4 (carbonic anhydrase 4), PRF1 (perforin 1), IL15 (interleukin 15), MYC, MMP2 (matrix metallopeptidase 2), SNAI2 (snail family transcriptional repressor 2), IFIT1 (interferon induced protein with tetratricopeptide repeats 1), EGR2 (early growth response 2), IL3RA (interleukin 3 receptor subunit alpha), CX3CL1 (C-X3-C motif chemokine ligand 1), EBI3 (Epstein-Barr virus induced 3), and ITK (IL2 inducible T cell kinase) were up-regulated, while the expression of XAGE1B (X antigen family member 1B) was down-regulated in early recurrent group than those in late recurrent group (Figure 2).

### The relationship of differentially expressive genes and the DFS time

**Figure 3:**
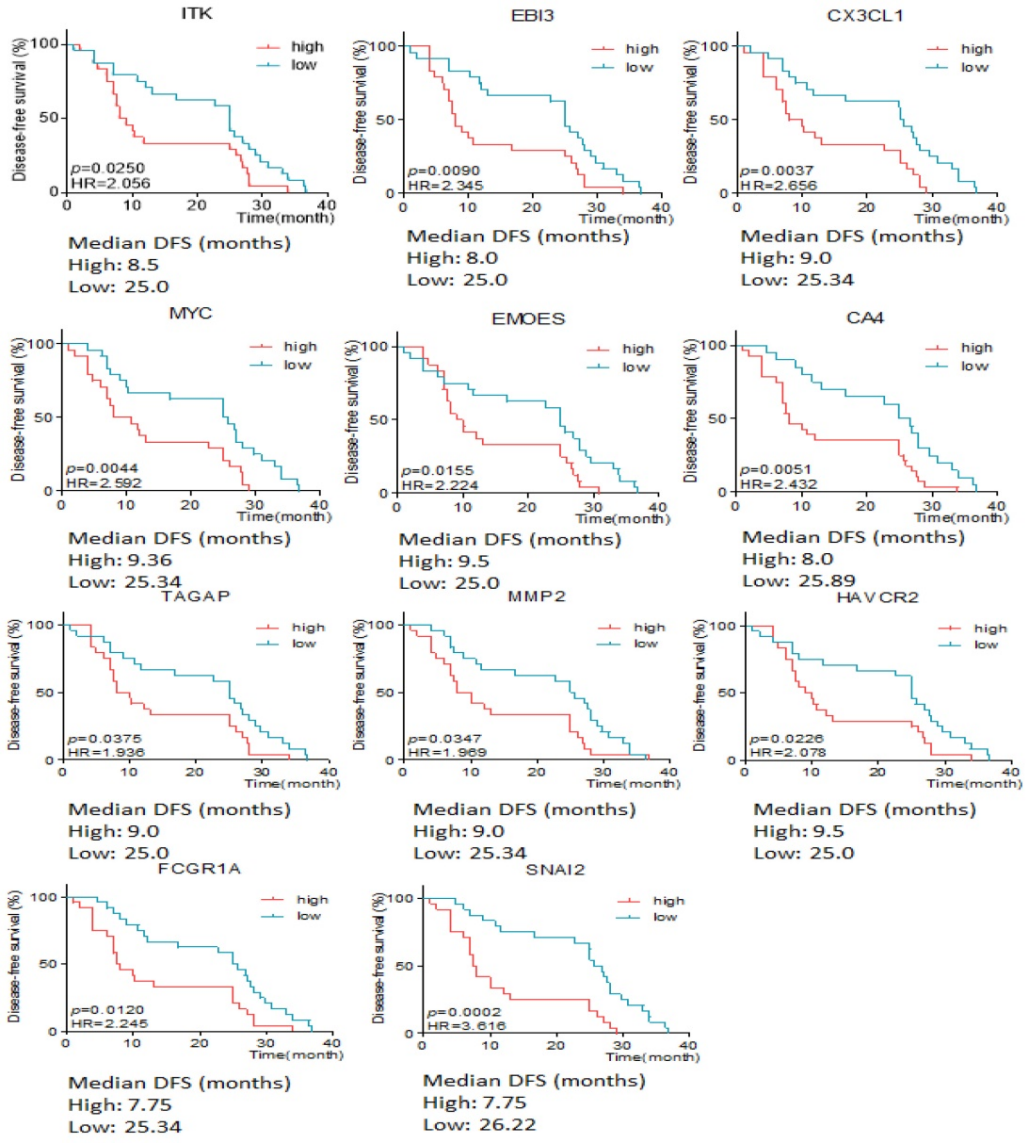
The disease free survival time according to the different expressive level of genes in early and late recurrent groups.

The association between differentially expressive genes and the survival time was analyzed by X-tile software basing on the data from RIOP sequencing. The results showed that high expression of ITK, EBI3, CX3CL1, MYC, EOMES, CA4, TAGAP, MMP2, HAVCR2, FCGR1 and SNAI2 predicted early recurrence by Gehan-Breslow-Wilcoxon test (*P*<0.05, Seen in Figure 3).

### The changes of immune-related RNA expression in different Lauren types

**Figure 4:**
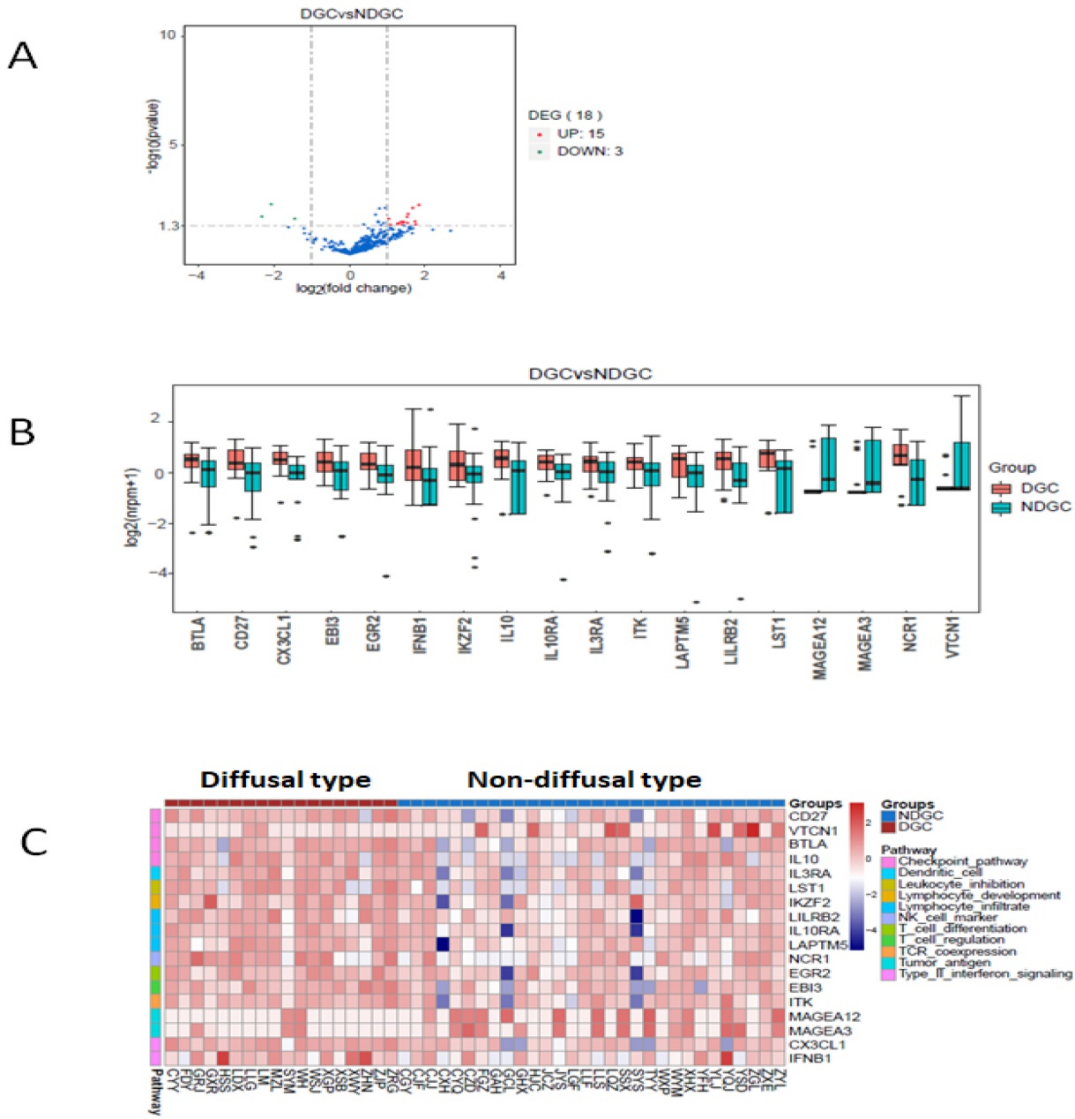
The enrichment analysis of genes' expression in diffusal type and non-diffusal type of gastric cancer. 15 genes were higher and 3 genes were lower expressed in diffusal type of gastric cancer (DGC) than that in non-diffusal type of gastric cancer (NDGC). A: The volcano plot. B: The box plot. C: The heatmap of cluster analysis.

**Figure 5:**
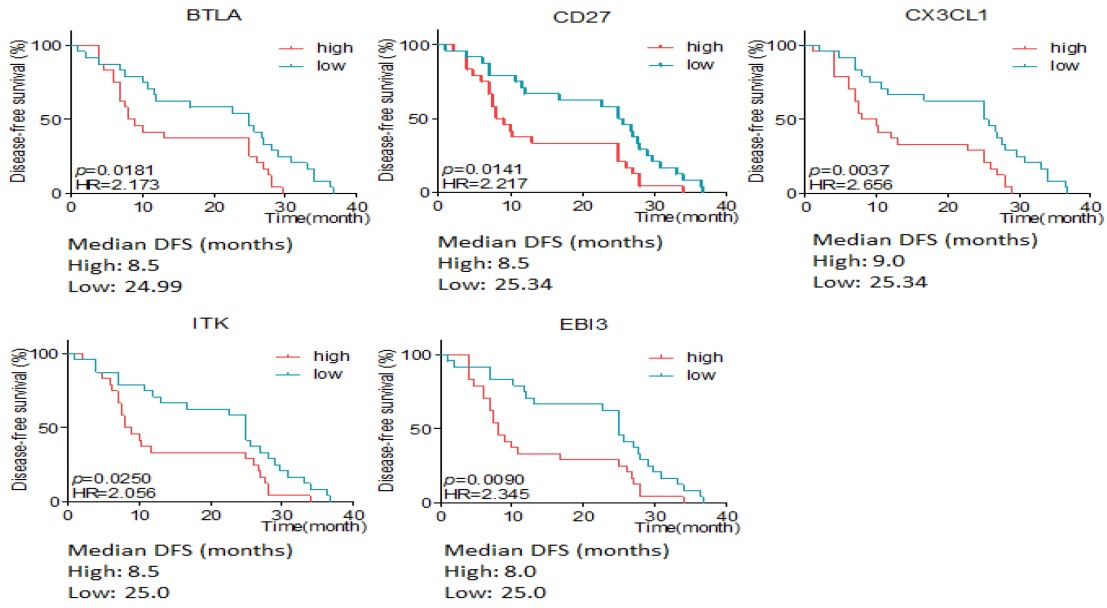
The disease free survival (DFS) time according to different expressive level of genes in diffusal and non-diffusal type of gastric cancer. High expression of BTLA, CD27, CX3CL1, ITK and EBI3 predicted short DFS time.

Here we defined intestinal type and mixed type to be non-diffusal type of GC. The DFS time was 13.48±2.12 and 20.02±2.15months in diffusal type (n=18) and non-diffusal type (n=30) of GC, respectively (*P*=0.04). There were 18 genes that showed significant difference between diffusal type and non-diffusal type of GC. The expression of CD27, BTLA (B and T lymphocyte associated), IL10 (interleukin 10), IL3RA (interleukin 3 receptor subunit alpha), IL10RA (interleukin 10 receptor subunit alpha), LST1 (leukocyte specific transcript 1), IKZF2 (IKAROS family zinc finger 2), LILRB2 (leukocyte immunoglobulin like receptor B2), LAPTM5 (lysosomal protein transmembrane 5), NCR1 (natural cytotoxicity triggering receptor 1), EGR2 (early growth response 2), CX3CL1 (C-X3-C motif chemokine ligand 1), EBI3 (Epstein-Barr virus induced 3), ITK (IL2 inducible T cell kinase), and IFNB1 (interferon beta 1) were observed to be higher in diffusal type than that in non-diffusal type of GC, while the expression of VTCN1 (V-set domain containing T cell activation inhibitor 1), MAGEA3 (MAGE Family Member A3), and MAGEA12 (MAGE Family Member A12) were detected to be lower in diffusal type than that in non-diffusal type of GC (Figure 4). High expression of BTLA, CD27, CX3CL1, ITK, and EBI3 predicted short DFS time and high rate of recurrence (Figure 5).

### Immune-related signal/functional pathway by cluster analysis in different groups

**Figure 6:**
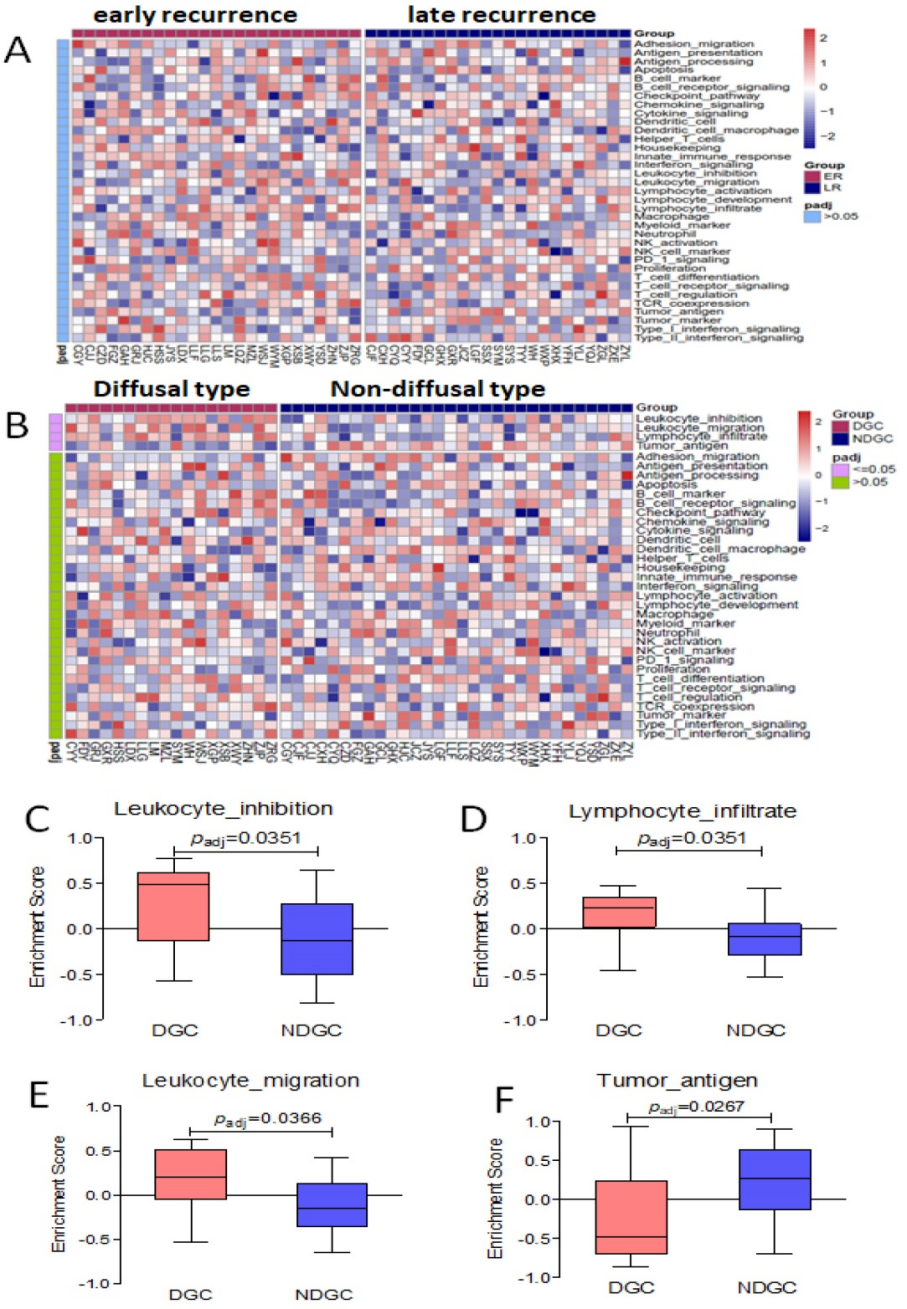
No different immune-related signal/functional pathway was found in early or late recurrent group. The enrichment score of Leukocyte-inhibition, Leukocyte-migration, and Lymphocyte-infiltrate signal/functional pathways were higher in diffusal type than that in non-diffusal type of GC, but the higher score of Tumor-antigen signal/functional pathway was seen in non-diffusal type. A: The heatmap of cluster analysis from 36 signal/functional pathways between early and late recurrent groups of gastric cancer. B: The heatmap of cluster analysis from 36 signal/functional pathways between diffusal type and non-diffusal type of gastric cancer. C: The difference of original enrichment score about Leukocyte-inhibition signal/functional pathway. D: The difference of original enrichment score about Leukocyte-migration signal/functional pathway. E: The difference of original enrichment score about Lymphocyte-infiltrate signal/functional pathway. F: The difference of original enrichment score about Tumor-antigen signal/functional pathway.

By cluster analysis of 36 immune-related signal/functional pathways including all of the 398 genes among eligible patients, we found that Leukocyte-inhibition, Leukocyte-migration, and Lymphocyte-infiltrate signal/functional pathway was associated with diffusal type of GC, and Tumor-antigen signal/functional pathways were activated in non-diffusal type of GC (*P*<0.05, Seen in Figure 6). However, we didn't find any significantly different signal/functional pathways between early and late recurrent groups.

### To explore the gene set that will predict the prognosis

**Figure 7:**
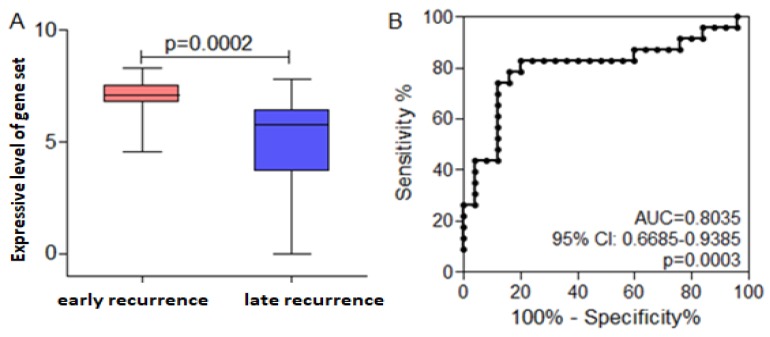
Receiver operating curve (ROC) analysis indicated the cut-off level of the gene set consisted of ITK, EBI3, and CX3CL1, which would predict early recurrence and poor prognosis. Early recurrence group was hypothesized to be the experimental one, and late recurrence group to be the control. A. The mean value of the gene set was much higher in early recurrent group than that in late recurrent group. B. High expressive level of the gene set could predict early recurrence and poor prognosis in gastric cancer basing on the ROC curve.

It is well-known that diffusal type of GC has poor prognosis, and is prone to metastasize early after radical resection. CX3CL1, EBI3, and ITK were not only higher expressive in early recurrent group than that in late recurrent group, but also expressed highly in diffusal type than that in non-diffusal type of GC. Then we used CX3CL1, EBI3, and ITK as a gene set to analyze whether it could predict the prognosis of GC. We found that the mean value of the gene set was much higher in early recurrent group than that in late recurrent group. By using ROC curve plotting, the expressive level of the gene set (AUC=0.8035; 95% CI: 0.668-0.9385; P=0.0003; sensitivity: 82.62%; speficity: 80%) could differentiate early or late recurrence. If the value was less than 6.66, the patient will not get early recurrence (Figure 7).

## Discussion

Despite the identification of numerous genes, mutant alleles and signaling networks as well as resistance mechanisms associated with cancer progression, GC remains a leading cause of death worldwide due to the limited efficacy of currently available treatment modalities. Most of the death of GC patients occur either due to relapse or metastasis via complex molecular mechanisms, where most of the conventional therapies failed. A suppressive immune TME has been considered as one of the hallmarks of GC, which plays a crucial role in tumor relapse or metastasis. For example, although PD-1/PD-L1 has emerged as a potential immune marker for prognosis and predictions in a range of malignancies, the overall response rate of anti-PD-1/PD-L1 treatment in GC was only about 12% 12-13 and some patients even developed hyper-progressive disease (HPD). Therefore, it is utmost urgent to find the key genes that not only predict the recurrent risk, but also can convert the tumor microenvironment from the immune-desert into the inflamed status.

Tumor recurrence has become a common characteristic feature of GC. Despite the remarkable achievement in the treatment strategies of GC, there is still high possibility of recurrence. The interaction of tumor cells with TME involves multiple signal and functional pathways and genes. However, with the advances and rapid progression in the new technologies such as NGS and RIOP, it is possible to investigate, analyze and identify the target genes/markers related to immune system in TME more efficiently and accurately in a comprehensive manner. In this study, we aimed to explore whether the immunological biomarkers at RNA level from the FFPE samples could predict the recurrent risk in locally advanced GC, even after radical resection. Therefore, we selected the detecting platform of RIOP including 398 genes 14.

The advantages of RIOP was high throughput and sensitive, and it could detect the low expressive cytokines at RNA level in FFPE samples. A predefined yield of 10ng of RNA and 30ng of DNA was used as acceptance criteria to ensure adequate library preparation^14^. The designed panel covered the signaling pathway of tumor immune response, immunological system activation, antigen presentation, immune cell differentiation, immune regulation, tumor antigens, and so on. Comparing with the technology of whole transcriptome sequencing, the detecting sensitivity of RIOP is 20 times high. Therefore, RIOP analysis can find the coding genes of low expressive cytokines in tumor cells and TME, and is able to detect the expression of different genes which are even lower than two times 15-18.

We detected the RNA expressive status of immunological biomarkers in FFPE slices of GC by RIOP. The results showed that the expression of ITK, EBI3, CX3CL1, MYC, EOMES, CA4, TAGAP, MMP2, HAVCR2, FCGR1 and SNAI2 were not only associated with early recurrence of GC after sugery, but also with the short DFS time. High expression of BTLA, CD27, CX3CL1, ITK, and EBI3 were seen in diffusal type of GC and predicted short DFS time. Therefore, it could be inferred that ITK, EBI3, and CX3CL1 were highly expressive both in early recurrent group and in diffusal type of GC. Then we used ITK, EBI3, and CX3CL1 as a gene set and verified that it could predict early recurrence and poor prognosis of GC by ROC curve plotting.

CX3CL1 was the only member in CX3C chemokine family, whose specific receptor was CX3CR1. CX3CL1/CX3CR1 axis played a major role in a wide range of biological process from adhesion to inflammation and cancer^19^. The overexpression of CX3CL1 and CX3CR1 in GC was associated with proliferation, metastasis and short survival time^20^, which was consistent with the result of our study. ITK was expressed in T cells, and played an important role in autoimmune inflammatory diseases through regulating the balance of Th17/Treg. It was reported that ITK was aberrantly expressed in melanoma and promoted tumor development and progression. ITK protein expression increased with nevus to metastatic melanoma progression 21. We found that ITK was relevant with early recurrence after curative resection in GC. EBI3 is a member of the IL-12 family structurally related to the subunit p40 of IL-12 and forms a heterodimer either with the p28 subunit to build IL-27 or with p35 to form IL-35 22. Interleukin-27 is secreted by antigen-presenting cells whereas IL-35 appears to be produced mainly by regulatory T cells and regulatory B cells, but both cytokines negatively regulate inflammatory immune responses 23-24. EBI3 was reported to be strongly related with larger tumor size and invasion depth in gastric cancer 25.

Among the 36 immune-related pathways, leukocyte-inhibition, leukocyte- migration, and lymphocyte-infiltrate signal/functional pathways in our RIOP were the top-ranking relevant ones by cluster analysis in diffusal type of GC, but tumor antigen signal/functional pathway was activated in non-diffusal type of GC. Combined with the genes in these pathways (Seen in Supplementary Table 1), we found that IL10RA, LST1, LILRB2, LAPTM5, and CX3CL1were verified to be higher expressive in diffusal type of GC than those in non-diffusal type of GC. However, the expression of MAGEA3 and MAGEA12 were detected to be lower in diffusal type than that in non-diffusal type of GC. Therefore, the carcinogenesis and development of diffusal type of gastric cancer, which easily gets peritoneal metastasis, might be mainly caused by immunosuppression, while the non-diffusal type of GC with good prognosis could have high mutation burden and sensitive to immunotherapy.

In summary, we are the first who used the highly sensitive RIOP to detect the RNA expressive status associated with recurrence or Lauren type in FFPE slices of locally advanced GC after surgery. We found that leukocyte-inhibition, leukocyte-migration, and lymphocyte-infiltrate signal/functional pathways were activated in diffuse type of GC. Furthermore, a validation study established a gene set consisted of ITK, EBI3, and CX3CL1 as potential indicators to design best treatment strategies for recurrent GC. Thus, these immune-related genes may provide potential targets for prediction of early recurrence or poor prognosis of GC patients, and some targets might become novel immune-therapy markers, which might add profound impact on the health status of GC patients in future.

## Supplementary Material

Supplementary figures and tables.

## Figures and Tables

**Table 1:** Patients' basic characteristic.

Characteristic		The number of patients	% (n=48)
**Gender**	Male	35	73%
	Female	13	27%
**Lauren's type**	Diffusal type	18	38%
	Non-diffusal type	30	62%
**Positive rate of lymph node**	High (≥50%)	23	48%
	Low (<50%)	25	52%
**Vascular cancerous thrombus**	Positive (+)	41	85%
	Negative (-)	7	15%
**Perineural invasion**	Positive (+)	38	79%
	Negative (-)	10	21%
**T stage**	T4	33	69%
	T3	13	27%
	T2	2	4%
**N stage**	N3	33	69%
	N2	14	29%
	N1	1	2%
**DFS**	Early recurrence	25	53%
	Late recurrence	23	47%
