# Supplementary material for: Identifying Predictive Factors of Recurrence after Radical Resection in Gastric Cancer by RNA Immune-oncology Panel
Source: J Cancer. 2020 Jan 1;11(3):638–47. doi: 10.7150/jca.38536 (PMC6959033; doi:10.7150/jca.38536)
Supplement: Supplementary file 1 — Supplementary figures and tables. [file jcav11p0638s1.pdf]

Supplementary Table 1. The list of the 398 genes.

NCBI Accession ID	Gene Symbol	Entrez ID	Gene name	Gene function
NM_078481	ADGRE5	976	adhesion G protein-coupled receptor E5	Adhesion,migration
NM_006566	CD226	10666	CD226 molecule	Adhesion,migration
NM_001775	CD38	952	CD38 molecule	Adhesion,migration
NM_000610	CD44	960	CD44 molecule Indian blood group	Adhesion,migration
NM_001777	CD47	961	CD47 molecule	Adhesion,migration
NM_001040033	CD53	963	CD53 molecule	Adhesion,migration
NM_000875	IGF1R	3480	insulin like growth factor 1 receptor	Adhesion,migration
NM_181501	ITGA1	3672	integrin subunit alpha 1	Adhesion,migration
NM_002208	ITGAE	3682	integrin subunit alpha E	Adhesion,migration
NM_002211	ITGB1	3688	integrin subunit beta 1	Adhesion,migration
NM_130760	MADCAM1	8174	mucosal vascular addressin cell adhesion molecule 1	Adhesion,migration
NM_181351	NCAM1	4684	neural cell adhesion molecule 1	Adhesion,migration
NM_001042724	NECTIN2	5819	nectin cell adhesion molecule 2	Adhesion,migration
NM_000442	PECAM1	5175	platelet and endothelial cell adhesion molecule 1	Adhesion,migration
NM_001765	CD1C	911	CD1c molecule	Antigen presentation
NM_001766	CD1D	912	CD1d molecule	Antigen presentation
NM_004233	CD83	9308	CD83 molecule	Antigen presentation
NM_001025159	CD74	972	CD74 molecule	Antigen processing

NM_002116	HLA-A	3105	major histocompatibility complex class I A	Antigen processing
NM_005514	HLA-B	3106	major histocompatibility complex class I B	Antigen processing
NM_002117	HLA-C	3107	major histocompatibility complex class I C	Antigen processing
NM_006120	HLA-DMA	3108	major histocompatibility complex class II DM alpha	Antigen processing
NM_002118	HLA-DMB	3109	major histocompatibility complex class II DM beta	Antigen processing
NM_002119	HLA-DOA	3111	major histocompatibility complex class II DO alpha	Antigen processing
NM_002120	HLA-DOB	3112	major histocompatibility complex class II DO beta	Antigen processing
NM_033554	HLA-DPA1	3113	major histocompatibility complex class II DP alpha 1	Antigen processing
NM_002121	HLA-DPB1	3115	major histocompatibility complex class II DP beta 1	Antigen processing
NM_002122	HLA-DQA1	3117	major histocompatibility complex class II DQ alpha 1	Antigen processing
NM_020056	HLA-DQA2	3118	major histocompatibility complex class II DQ alpha 2	Antigen processing
NM_001198858	HLA-DQB2	3120	major histocompatibility complex class II DQ beta 2	Antigen processing
NM_019111	HLA-DRA	3122	major histocompatibility complex class II DR alpha	Antigen processing

NM_002124	HLA-DRB1	3123	major histocompatibility complex class II DR beta 1	Antigen processing
NM_005516	HLA-E	3133	major histocompatibility complex class I E	Antigen processing
NM_001098479	HLA-F	3134	major histocompatibility complex class I F	Antigen processing
NR_026972	HLA-F-AS1	285830	HLA-F antisense RNA 1	Antigen processing
NM_002127	HLA-G	3135	major histocompatibility complex class I G	Antigen processing
NM_000633	BCL2	596	B-cell CLL lymphoma 2	Apoptosis
NM_138621	BCL2L11	10018	BCL2 like 11	Apoptosis
NM_052850	GADD45GIP1	90480	GADD45G interacting protein 1	Apoptosis
NM_003810	TNFSF10	8743	tumor necrosis factor superfamily member 10	Apoptosis
NM_001178098	CD19	930	CD19 molecule	B cell marker
NM_001771	CD22	933	CD22 molecule	B cell marker
NM_000566	FCGR1A	22,092,210	Fc fragment of IgG receptor Ia	B cell marker
NM_004001	FCGR2B	2213	Fc fragment of IgG receptor IIb	B cell marker
NM_001184866	FCRLA	84824	Fc receptor like A	B cell marker
NM_144646	JCHAIN	3512	joining chain of multimeric IgA and IgM	B cell marker
NM_006181	NTN3	4917	netrin 3	B cell marker
NM_006235	POU2AF1	5450	POU class 2 associating factor 1	B cell marker
NM_003930	SKAP2	8935	src kinase associated phosphoprotein 2	B cell marker
NM_001192	TNFRSF17	608	tumor necrosis factor receptor	B cell marker

			superfamily member 17	
NM_006573	TNFSF13B	10673	tumor necrosis factor superfamily member 13b	B cell marker
NM_001039933	CD79B	974	CD79b molecule	B cell receptor signaling
NM_001783	CD79A	973	CD79a molecule	B cell receptor signaling
NM_000043	FAS	355	Fas cell surface death receptor	B cell receptor signaling
NM_000675	ADORA2A	135	adenosine A2a receptor	Checkpoint pathway
NM_181780	BTLA	151888	B and T lymphocyte associated	Checkpoint pathway
NM_022153	C10orf54	64115	chromosome 10 open reading frame 54	Checkpoint pathway
NM_007053	CD160	11126	CD160 molecule	Checkpoint pathway
NM_001166663	CD244	51744	CD244 molecule	Checkpoint pathway
NM_014143	CD274	29126	CD274 molecule	Checkpoint pathway
NM_001024736	CD276	80381	CD276 molecule	Checkpoint pathway
NM_006139	CD28	940	CD28 molecule	Checkpoint pathway
NM_001778	CD48	962	CD48 molecule	Checkpoint pathway
NM_001781	CD69	969	CD69 molecule	Checkpoint pathway
NM_005191	CD80	941	CD80 molecule	Checkpoint pathway
NM_175862	CD86	942	CD86 molecule	Checkpoint pathway
NM_001712	CEACAM1	634	carcinoembryonic antigen related cell adhesion molecule 1	Checkpoint pathway
NM_001098175	ENTPD1	953	ectonucleoside triphosphate diphosphohydrolase 1	Checkpoint pathway
NM_005442	EOMES	8320	eomesodermin	Checkpoint pathway
NM_032782	HAVCR2	84868	hepatitis A virus cellular receptor 2	Checkpoint pathway

NM_012092	ICOS	29851	inducible T-cell costimulator	Checkpoint pathway
NM_015259	ICOSLG	23308	inducible T-cell costimulator ligand	Checkpoint pathway
NM_194294	IDO2	169355	indoleamine 23-dioxygenase 2	Checkpoint pathway
NM_002526	NT5E	4907	5-nucleotidase ecto	Checkpoint pathway
NM_025239	PDCD1LG2	80380	programmed cell death 1 ligand 2	Checkpoint pathway
NM_006505	PVR	5817	poliovirus receptor	Checkpoint pathway
NM_005651	TDO2	6999	tryptophan 23-dioxygenase	Checkpoint pathway
NM_000660	TGFB1	7040	transforming growth factor beta 1	Checkpoint pathway
NM_000594	TNF	7124	tumor necrosis factor	Checkpoint pathway
NM_003820	TNFRSF14	8764	tumor necrosis factor receptor superfamily member 14	Checkpoint pathway
NM_003807	TNFSF14	8740	tumor necrosis factor superfamily member 14	Checkpoint pathway
NM_005092	TNFSF18	8995	tumor necrosis factor superfamily member 18	Checkpoint pathway
NM_003326	TNFSF4	7292	tumor necrosis factor superfamily member 4	Checkpoint pathway
NM_024626	VTCN1	79679	V-set domain containing T cell activation inhibitor 1	Checkpoint pathway
NM_002987	CCL17	6361	C-C motif chemokine ligand 17	Chemokine signaling
NM_004591	CCL20	6364	C-C motif chemokine ligand 20	Chemokine signaling
NM_002990	CCL22	6367	C-C motif chemokine ligand 22	Chemokine signaling
NM_005508	CCR4	1233	C-C motif chemokine receptor 4	Chemokine signaling
NM_004367	CCR6	1235	C-C motif chemokine receptor 6	Chemokine signaling
NM_001511	CXCL1	2919	C-X-C motif chemokine ligand 1	Chemokine signaling

NM_001557	CXCR2	3579	C-X-C motif chemokine receptor 2	Chemokine signaling
NM_001504	CXCR3	2833	C-X-C motif chemokine receptor 3	Chemokine signaling
NM_000265	NCF1	653361	neutrophil cytosolic factor 1	Chemokine signaling
NM_001171623	VEGFA	7422	vascular endothelial growth factor A	Chemokine signaling
NM_001295	CCR1	1230	C-C motif chemokine receptor 1	Cytokine signaling
NM_000395	CSF2RB	1439	colony stimulating factor 2 receptor beta common subunit	Cytokine signaling
NM_000584	CXCL8	3576	C-X-C motif chemokine ligand 8	Cytokine signaling
NM_000601	HGF	3082	hepatocyte growth factor	Cytokine signaling
NM_001547	IFIT2	3433	interferon induced protein with tetratricopeptide repeats 2	Cytokine signaling
NM_002188	IL13	3596	interleukin 13	Cytokine signaling
NM_000575	IL1A	3552	interleukin 1 alpha	Cytokine signaling
NM_021803	IL21	59067	interleukin 21	Cytokine signaling
NM_000589	IL4	3565	interleukin 4	Cytokine signaling
NM_000600	IL6	3569	interleukin 6	Cytokine signaling
NM_000880	IL7	3574	interleukin 7	Cytokine signaling
NM_003811	TNFSF9	8744	tumor necrosis factor superfamily member 9	Cytokine signaling
NM_005211	CSF1R	1436	colony stimulating factor 1 receptor	Cytokine signaling
NM_000417	IL2RA	3559	interleukin 2 receptor subunit alpha	Cytokine signaling
NM_003152	STAT5A	6776	signal transducer and activator of transcription 5A	Cytokine signaling
NM_130441	CLEC4C	170482	C-type lectin domain family 4	Dendridic cell

			member C	
NM_017912	HERC6	55008	HECT and RLD domain containing E3 ubiquitin protein ligase family mem	Dendridic cell
NM_002183	IL3RA	3563	interleukin 3 receptor subunit alpha	Dendridic cell
NM_000887	ITGAX	3687	integrin subunit alpha X	Dendridic cell
NM_003873	NRP1	8829	neuropilin 1	Dendridic cell
NM_003265	TLR3	7098	toll like receptor 3	Dendridic cell
NM_025224	ZBTB46	140685	zinc finger and BTB domain containing 46	Dendridic cell
NM_000591	CD14	929	CD14 molecule	Dendridic cell,macrophage
NM_021155	CD209	30835	CD209 molecule	Dendridic cell,macrophage
NM_001142345	CMKLR1	1240	chemerin chemokine-like receptor 1	Dendridic cell,macrophage
NM_052872	IL17F	112744	interleukin 17F	Dendridic cell,macrophage
NM_016584	IL23A	51561	interleukin 23 subunit alpha	Dendridic cell,macrophage
NM_002438	MRC1	4360	mannose receptor C type 1	Dendridic cell,macrophage
NM_001242	CD27	939	CD27 molecule	Drug target
NM_001250	CD40	958	CD40 molecule	Drug target
NM_001252	CD70	970	CD70 molecule	Drug target

NM_005214	CTLA4	1493	cytotoxic T-lymphocyte associated protein 4	Drug target
NM_002164	IDO1	3620	indoleamine 23-dioxygenase 1	Drug target
NM_000572	IL10	3586	interleukin 10	Drug target
NM_000882	IL12A	3592	interleukin 12A	Drug target
NM_002187	IL12B	3593	interleukin 12B	Drug target
NM_000586	IL2	3558	interleukin 2	Drug target
NM_014218	KIR2DL1	3802	killer cell immunoglobulin like receptor two Ig domains and long cytopla	Drug target
NM_002262	KLRD1	3824	killer cell lectin like receptor D1	Drug target
NM_002286	LAG3	3902	lymphocyte activating 3	Drug target
NM_021950	MS4A1	931	membrane spanning 4-domains A1	Drug target
NM_005018	PDCD1	5133	programmed cell death 1	Drug target
NM_006928	PMEL	6490	premelanosome protein	Drug target
NM_021181	SLAMF7	57823	SLAM family member 7	Drug target
NM_139276	STAT3	6774	signal transducer and activator of transcription 3	Drug target
NM_017442	TLR9	54106	toll like receptor 9	Drug target
NM_004195	TNFRSF18	8784	tumor necrosis factor receptor superfamily member 18	Drug target
NM_003327	TNFRSF4	7293	tumor necrosis factor receptor superfamily member 4	Drug target
NM_001561	TNFRSF9	3604	tumor necrosis factor receptor superfamily member 9	Drug target

NM_006399	BATF	10538	basic leucine zipper ATF-like transcription factor	Helper T cells
NM_001123396	CCR2	729230	C-C motif chemokine receptor 2	Helper T cells
NM_000616	CD4	920	CD4 molecule	Helper T cells
NM_001002295	GATA3	2625	GATA binding protein 3	Helper T cells
NM_002190	IL17A	3605	interleukin 17A	Helper T cells
NM_005060	RORC	6097	RAR related orphan receptor C	Helper T cells
NM_003151	STAT4	6775	signal transducer and activator of transcription 4	Helper T cells
NM_003153	STAT6	6778	signal transducer and activator of transcription 6	Helper T cells
NM_001025091	ABCF1	23	ATP binding cassette subfamily F member 1	Housekeeping
NM_000402	G6PD	2539	glucose-6-phosphate dehydrogenase	Housekeeping
NM_000181	GUSB	2990	glucuronidase beta	Housekeeping
NM_000190	HMBS	3145	hydroxymethylbilane synthase	Housekeeping
NM_170707	LMNA	4000	lamin A C	Housekeeping
NM_002332	LRP1	4035	LDL receptor related protein 1	Housekeeping
NM_000937	POLR2A	5430	polymerase RNA II subunit A	Housekeeping
NM_004168	SDHA	6389	succinate dehydrogenase complex flavoprotein subunit A	Housekeeping
NM_003194	TBP	6908	TATA-box binding protein	Housekeeping
NM_001128148	TFRC	7037	transferrin receptor	Housekeeping
NM_178014	TUBB	203068	tubulin beta class I	Housekeeping
NM_021913	AXL	558	AXL receptor tyrosine kinase	Innate immune response

NM_015991	C1QA	712	complement component 1 q subcomponent A chain	Innate immune response
NM_007329	DMBT1	1755	deleted in malignant brain tumors 1	Innate immune response
NM_022168	IFIH1	64135	interferon induced with helicase C domain 1	Innate immune response
NM_000625	NOS2	4843	nitric oxide synthase 2	Innate immune response
NM_016562	TLR7	51284	toll like receptor 7	Innate immune response
NM_000491	C1QB	713	complement component 1 q subcomponent B chain	Innate immune response
NM_005564	LCN2	3934	lipocalin 2	Innate immune response
NM_000239	LYZ	4069	lysozyme	Innate immune response
NM_139012	MAPK14	1432	mitogen-activated protein kinase 14	Innate immune response
NM_002415	MIF	4282	macrophage migration inhibitory factor glycosylation-inhibiting factor	Innate immune response
NM_014314	DDX58	23586	DEXD H-box helicase 58	Interferon signaling
NM_005533	IFI35	3430	interferon induced protein 35	Interferon signaling
NM_006820	IFI44L	10964	interferon induced protein 44 like	Interferon signaling
NM_022873	IFI6	2537	interferon alpha inducible protein 6	Interferon signaling
NM_002460	IRF4	3662	interferon regulatory factor 4	Interferon signaling
NM_001178046	MX1	4599	MX dynamin like GTPase 1	Interferon signaling
NM_016817	OAS2	4939	2-5-oligoadenylate synthetase 2	Interferon signaling
NM_006187	OAS3	4940	2-5-oligoadenylate synthetase 3	Interferon signaling
NM_001081637	LILRB1	10859	leukocyte immunoglobulin like receptor B1	Leukocyte inhibition

NM_007161	LST1	7940	leukocyte specific transcript 1	Leukocyte inhibition
NM_002209	ITGAL	3683	integrin subunit alpha L	Leukocyte migration
NM_001145808	ITGAM	3684	integrin subunit alpha M	Leukocyte migration
NM_000889	ITGB7	3695	integrin subunit beta 7	Leukocyte migration
NM_000655	SELL	6402	selectin L	Leukocyte migration
NM_001078	VCAM1	7412	vascular cell adhesion molecule 1	Leukocyte migration
NM_002351	SH2D1A	4068	SH2 domain containing 1A	Lymphocyte activation
NM_053282	SH2D1B	117157	SH2 domain containing 1B	Lymphocyte activation
NM_006060	IKZF1	10320	IKAROS family zinc finger 1	Lymphocyte development
NM_016260	IKZF2	22807	IKAROS family zinc finger 2	Lymphocyte development
NM_022465	IKZF4	64375	IKAROS family zinc finger 4	Lymphocyte development
NM_002988	CCL18	6362	C-C motif chemokine ligand 18	Lymphocyte infiltrate
NM_002982	CCL2	6347	C-C motif chemokine ligand 2	Lymphocyte infiltrate
NM_002989	CCL21	6366	C-C motif chemokine ligand 21	Lymphocyte infiltrate
NM_002983	CCL3	6348	C-C motif chemokine ligand 3	Lymphocyte infiltrate
NM_002984	CCL4	6351	C-C motif chemokine ligand 4	Lymphocyte infiltrate
NM_002985	CCL5	6352	C-C motif chemokine ligand 5	Lymphocyte infiltrate
NM_001100168	CCR5	1234	C-C motif chemokine receptor 5 gene pseudogene	Lymphocyte infiltrate
NM_001767	CD2	914	CD2 molecule	Lymphocyte infiltrate
NM_001774	CD37	951	CD37 molecule	Lymphocyte infiltrate
NM_001803	CD52	1043	CD52 molecule	Lymphocyte infiltrate
NM_001780	CD63	967	CD63 molecule	Lymphocyte infiltrate
NM_007074	CORO1A	11151	coronin 1A	Lymphocyte infiltrate
NM_004079	CTSS	1520	cathepsin S	Lymphocyte infiltrate

ENST00000399220	CX3CR1	1524	C-X3-C motif chemokine receptor 1	Lymphocyte infiltrate
ENST00000435290	CX3CR1	1524	C-X3-C motif chemokine receptor 1	Lymphocyte infiltrate
ENST00000541347	CX3CR1	1524	C-X3-C motif chemokine receptor 1	Lymphocyte infiltrate
NM_001171174	CX3CR1	1524	C-X3-C motif chemokine receptor 1	Lymphocyte infiltrate
NM_003467	CXCR4	7852	C-X-C motif chemokine receptor 4	Lymphocyte infiltrate
NM_006564	CXCR6	10663	C-X-C motif chemokine receptor 6	Lymphocyte infiltrate
NM_004106	FCER1G	2207	Fc fragment of IgE receptor Ig	Lymphocyte infiltrate
NM_001465	FYB	2533	FYN binding protein	Lymphocyte infiltrate
NM_006144	GZMA	3001	granzyme A	Lymphocyte infiltrate
NM_004131	GZMB	3002	granzyme B	Lymphocyte infiltrate
NM_033423	GZMH	2999	granzyme H	Lymphocyte infiltrate
NM_002104	GZMK	3003	granzyme K	Lymphocyte infiltrate
NM_005849	IGSF6	10261	immunoglobulin superfamily member 6	Lymphocyte infiltrate
NM_001558	IL10RA	3587	interleukin 10 receptor subunit alpha	Lymphocyte infiltrate
NM_000206	IL2RG	3561	interleukin 2 receptor subunit gamma	Lymphocyte infiltrate
NM_000211	ITGB2	3689	integrin subunit beta 2	Lymphocyte infiltrate
NM_001098526	JAML	120425	junction adhesion molecule like	Lymphocyte infiltrate
NM_005561	LAMP1	3916	lysosomal associated membrane protein 1	Lymphocyte infiltrate
NM_006762	LAPTM5	7805	lysosomal protein transmembrane 5	Lymphocyte infiltrate
NM_001080978	LILRB2	10288	leukocyte immunoglobulin like	Lymphocyte infiltrate

			receptor B2	
NM_002348	LY9	4063	lymphocyte antigen 9	Lymphocyte infiltrate
NM_005601	NKG7	4818	natural killer cell granule protein 7	Lymphocyte infiltrate
NM_001199797	PTPN7	5778	protein tyrosine phosphatase non-receptor type 7	Lymphocyte infiltrate
NM_002838	PTPRC	5788	protein tyrosine phosphatase receptor type C	Lymphocyte infiltrate
NM_015474	SAMHD1	25939	SAM and HD domain containing deoxynucleoside triphosphate triphosph	Lymphocyte infiltrate
NM_014450	SIT1	27240	signaling threshold regulating transmembrane adaptor 1	Lymphocyte infiltrate
NM_020125	SLAMF8	56833	SLAM family member 8	Lymphocyte infiltrate
NM_002727	SRGN	5552	serglycin	Lymphocyte infiltrate
NM_054114	TAGAP	117289	T-cell activation RhoGTPase activating protein	Lymphocyte infiltrate
NM_001003806	TARP	445347	TCR gamma alternate reading frame protein	Lymphocyte infiltrate
NM_138636	TLR8	51311	toll like receptor 8	Lymphocyte infiltrate
NM_014350	TNFAIP8	25816	TNF alpha induced protein 8	Lymphocyte infiltrate
NM_198125	TYROBP	7305	TYRO protein tyrosine kinase binding protein	Lymphocyte infiltrate
NM_001623	AIF1	199	allograft inflammatory factor 1	Macrophage
NM_001141	ALOX15B	247	arachidonate 15-lipoxygenase type B	Macrophage
NM_004244	CD163	9332	CD163 molecule	Macrophage
NM_001251	CD68	968	CD68 molecule	Macrophage

NM_000569	FCGR3A	2214	Fc fragment of IgG receptor IIIa	Macrophage
NM_000045	ARG1	383	arginase 1	Myeloid marker
NM_001772	CD33	945	CD33 molecule	Myeloid marker
NM_001816	CEACAM8	1088	carcinoembryonic antigen related cell adhesion molecule 8	Myeloid marker
NM_000250	MPO	4353	myeloperoxidase	Myeloid marker
NM_002964	S100A8	6279	S100 calcium binding protein A8	Myeloid marker,MDSC
NM_002965	S100A9	6280	S100 calcium binding protein A9	Myeloid marker,MDSC
NM_002033	FUT4	2526	fucosyltransferase 4	Myeloid marker,stem cell
NM_000717	CA4	762	carbonic anhydrase 4	Neutrophil
NM_032564	DGAT2	84649	diacylglycerol O-acyltransferase 2	Neutrophil
NM_032045	KREMEN1	83999	kringle containing transmembrane protein 1	Neutrophil
NM_052972	LRG1	116844	leucine rich alpha-2-glycoprotein 1	Neutrophil
NM_002863	PYGL	5836	phosphorylase glycogen liver	Neutrophil
NM_018644	B3GAT1	27087	beta-13-glucuronyltransferase 1	NK activation
NM_000570	FCGR3B	2215	Fc fragment of IgG receptor IIIb	NK activation
NM_006433	GNLY	10578	granulysin	NK activation
NM_002258	KLRB1	3820	killer cell lectin like receptor B1	NK activation
NM_016523	KLRF1	51348	killer cell lectin like receptor F1	NK activation
NM_005810	KLRG1	10219	killer cell lectin like receptor G1	NK activation
NM_007360	KLRK1	22914	killer cell lectin like receptor K1	NK activation
NM_005041	PRF1	5551	perforin 1	NK activation
ENST00000344867	KIR2DL2	3803	killer cell immunoglobulin like receptor two Ig domains and long	NK cell marker

			cytopla	
NM_015868	KIR2DL3	3804	killer cell immunoglobulin like receptor two Ig domains and long cytopla	NK cell marker
NM_004829	NCR1	9437	natural cytotoxicity triggering receptor 1	NK cell marker
NM_147130	NCR3	259197	natural cytotoxicity triggering receptor 3	NK cell marker
NM_172387	NFATC1	4772	nuclear factor of activated T-cells 1	PD-1 signaling
NM_001198	PRDM1	639	PR domain 1	PD-1 signaling
NM_002015	FOXO1	2308	forkhead box O1	PD-1 signaling,tumor marker
NM_001530	HIF1A	3091	hypoxia inducible factor 1 alpha subunit	PD-1 signaling,tumor marker
NM_004958	MTOR	2475	mechanistic target of rapamycin	PD-1 signaling,tumor marker
NM_006218	PIK3CA	5290	phosphatidylinositol-45-bisphosphate 3-kinase catalytic subunit alpha	PD-1 signaling,tumor marker
NM_005026	PIK3CD	5293	phosphatidylinositol-45-bisphosphate 3-kinase catalytic subunit delta	PD-1 signaling,tumor marker
NM_000314	PTEN	5728	phosphatase and tensin homolog	PD-1 signaling,tumor marker
NM_002834	PTPN11	5781	protein tyrosine phosphatase non-receptor type 11	PD-1 signaling,tumor marker
NM_004336	BUB1	699	BUB1 mitotic checkpoint serine threonine kinase	Proliferation

NM_004701	CCNB2	9133	cyclin B2	Proliferation
NM_001786	CDK1	983	cyclin-dependent kinase 1	Proliferation
NM_005192	CDKN3	1033	cyclin-dependent kinase inhibitor 3	Proliferation
NM_021953	FOXM1	2305	forkhead box M1	Proliferation
NM_014736	KIAA0101	9768	KIAA0101	Proliferation
NM_002358	MAD2L1	4085	MAD2 mitotic arrest deficient-like 1 yeast	Proliferation
NM_014791	MELK	9833	maternal embryonic leucine zipper kinase	Proliferation
NM_002417	MKI67	4288	marker of proliferation Ki-67	Proliferation
NM_001067	TOP2A	7153	topoisomerase DNA II alpha	Proliferation
NM_000399	EGR2	1959	early growth response 2	T cell differentiation
NM_001110533	LEXM	163747	lymphocyte expansion molecule	T cell differentiation
NM_170662	CBLB	868	Cbl proto-oncogene B	T cell receptor signaling
NM_000074	CD40LG	959	CD40 ligand	T cell receptor signaling
NM_021268	IFNA17	3451	interferon alpha 17	T cell receptor signaling
NM_020529	NFKBIA	4792	NFKB inhibitor alpha	T cell receptor signaling
NM_080548	PTPN6	5777	protein tyrosine phosphatase non-receptor type 6	T cell receptor signaling
NM_001079	ZAP70	7535	zeta chain of T cell receptor associated protein kinase 70kDa	T cell receptor signaling
NM_005755	EBI3	10148	Epstein-Barr virus induced 3	T cell regulation
NM_014009	FOXP3	50943	forkhead box P3	T cell regulation
NM_002166	ID2	3398	inhibitor of DNA binding 2 HLH protein	T cell regulation

NM_002167	ID3	3399	inhibitor of DNA binding 3 HLH protein	T cell regulation
NM_000585	IL15	3600	interleukin 15	T cell regulation
NM_001562	IL18	3606	interleukin 18	T cell regulation
NM_020525	IL22	50616	interleukin 22	T cell regulation
NM_002355	M6PR	4074	mannose-6-phosphate receptor cation dependent	T cell regulation
NM_016270	KLF2	10365	Kruppel like factor 2	T cell regulation,trafficking
NM_001838	CCR7	1236	C-C motif chemokine receptor 7	TCR coexpression
NM_198053	CD247	919	CD247 molecule	TCR coexpression
NM_000732	CD3D	915	CD3d molecule	TCR coexpression
NM_000733	CD3E	916	CD3e molecule	TCR coexpression
NM_000073	CD3G	917	CD3g molecule	TCR coexpression
NM_006725	CD6	923	CD6 molecule	TCR coexpression
NM_171827	CD8A	925	CD8a molecule	TCR coexpression
NM_172213	CD8B	926	CD8b molecule	TCR coexpression
NM_019604	CRTAM	56253	cytotoxic and regulatory T-cell molecule	TCR coexpression
NM_001098200	GPR18	2841	G protein-coupled receptor 18	TCR coexpression
NM_004810	GRAP2	9402	GRB2-related adaptor protein 2	TCR coexpression
NM_012481	IKZF3	22806	IKAROS family zinc finger 3	TCR coexpression
NM_000878	IL2RB	3560	interleukin 2 receptor subunit beta	TCR coexpression
NM_002185	IL7R	3575	interleukin 7 receptor	TCR coexpression
NM_005546	ITK	3702	IL2 inducible T-cell kinase	TCR coexpression
NM_014398	LAMP3	27074	lysosomal associated membrane	TCR coexpression

			protein 3	
NM_001042771	LCK	3932	LCK proto-oncogene Src family tyrosine kinase	TCR coexpression
ENST00000326294	PTPRCAP	5790	protein tyrosine phosphatase receptor type C associated protein	TCR coexpression
NM_173799	TIGIT	201633	T-cell immunoreceptor with Ig and ITIM domains	TCR coexpression
NM_182482	BAGE	574	B melanoma antigen	Tumor antigen
NM_001327	CTAG1B	1485	cancer testis antigen 1B	Tumor antigen
ENST00000369585	CTAG2	30848	cancer testis antigen 2	Tumor antigen
NM_001040663	GAGE1,GAGE12I,GAGE12F	################	G antigen 1	Tumor antigen
NM_001098413	GAGE10	102724473	G antigen 10	Tumor antigen
NM_001098406	GAGE12J	729396	G antigen 12J	Tumor antigen
NM_001098412	GAGE13	645051	G antigen 13	Tumor antigen
NM_001472	GAGE2C,GAGE2A,GAGE2E	################	G antigen 2C	Tumor antigen
NM_004988	MAGEA1	4100	MAGE family member A1	Tumor antigen
NM_021048	MAGEA10	4109	MAGE family member A10	Tumor antigen
NM_005367	MAGEA12	4111	MAGE family member A12	Tumor antigen
NM_005362	MAGEA3	41,024,105	MAGE family member A3	Tumor antigen
NM_001011548	MAGEA4	4103	MAGE family member A4	Tumor antigen
NM_016249	MAGEC2	51438	MAGE family member C2	Tumor antigen
NM_005511	MLANA	2315	melan-A	Tumor antigen
NM_003147	SSX2	6757	SSX family member 2	Tumor antigen
NM_001097594	XAGE1B	653220	X antigen family member 1B	Tumor antigen

NM_001014431	AKT1	207	AKT serine threonine kinase 1	Tumor marker
NM_007300	BRCA1	672	BRCA1 DNA repair associated	Tumor marker
NM_000059	BRCA2	675	BRCA2 DNA repair associated	Tumor marker
NM_000077	CDKN2A	1029	cyclin-dependent kinase inhibitor 2A	Tumor marker
NM_005227	EFNA4	1945	ephrin A4	Tumor marker
NM_005228	EGFR	1956	epidermal growth factor receptor	Tumor marker
NM_004430	EGR3	1960	early growth response 3	Tumor marker
NM_005544	IRS1	3667	insulin receptor substrate 1	Tumor marker
NM_000424	KRT5	3852	keratin 5	Tumor marker
NM_005556	KRT7	3855	keratin 7	Tumor marker
NM_002745	MAPK1	5594	mitogen-activated protein kinase 1	Tumor marker
NM_004530	MMP2	4313	matrix metallopeptidase 2	Tumor marker
NM_004994	MMP9	4318	matrix metallopeptidase 9	Tumor marker
NM_002467	MYC	4609	v-myc avian myelocytomatosis viral oncogene homolog	Tumor marker
NM_000435	NOTCH3	4854	notch 3	Tumor marker
NM_002632	PGF	5228	placental growth factor	Tumor marker
NM_000963	PTGS2	5743	prostaglandin-endoperoxide synthase 2	Tumor marker
NM_002821	PTK7	5754	protein tyrosine kinase 7 inactive	Tumor marker
NM_000321	RB1	5925	RB transcriptional corepressor 1	Tumor marker
NM_001010	RPS6	6194	ribosomal protein S6	Tumor marker
NM_003202	TCF7	6932	transcription factor 7 T-cell specific HMG-box	Tumor marker

NM_003722	TP63	8626	tumor protein p63	Tumor marker
NM_012101	TRIM29	23650	tripartite motif containing 29	Tumor marker
NM_005985	SNAI1	6615	snail family transcriptional repressor 1	Tumor marker,stemness
NM_003068	SNAI2	6591	snail family transcriptional repressor 2	Tumor marker,stemness
NM_000474	TWIST1	7291	twist family bHLH transcription factor 1	Tumor marker,stemness
NM_001174093	ZEB1	6935	zinc finger E-box binding homeobox 1	Tumor marker,stemness
NM_004335	BST2	684	bone marrow stromal cell antigen 2	Type I interferon signaling
NM_005532	IFI27	3429	interferon alpha inducible protein 27	Type I interferon signaling
NM_001548	IFIT1	3434	interferon induced protein with tetratricopeptide repeats 1	Type I interferon signaling
NM_001031683	IFIT3	3437	interferon induced protein with tetratricopeptide repeats 3	Type I interferon signaling
NM_003641	IFITM1	8519	interferon induced transmembrane protein 1	Type I interferon signaling
NM_006435	IFITM2	10581	interferon induced transmembrane protein 2	Type I interferon signaling
NM_005101	ISG15	9636	ISG15 ubiquitin-like modifier	Type I interferon signaling
NM_002201	ISG20	3669	interferon stimulated exonuclease gene 20	Type I interferon signaling
NM_001706	BCL6	604	B-cell CLL lymphoma 6	Type II interferon signaling

NM_000246	CIITA	4261	class II major histocompatibility complex transactivator	Type II interferon signaling
NM_002996	CX3CL1	6376	C-X3-C motif chemokine ligand 1	Type II interferon signaling
NM_001565	CXCL10	3627	C-X-C motif chemokine ligand 10	Type II interferon signaling
NM_005409	CXCL11	6373	C-X-C motif chemokine ligand 11	Type II interferon signaling
NM_006419	CXCL13	10563	C-X-C motif chemokine ligand 13	Type II interferon signaling
NM_002416	CXCL9	4283	C-X-C motif chemokine ligand 9	Type II interferon signaling
NM_001716	CXCR5	643	C-X-C motif chemokine receptor 5	Type II interferon signaling
NM_000397	CYBB	1536	cytochrome b-245 beta chain	Type II interferon signaling
NM_001135651	EIF2AK2	5610	eukaryotic translation initiation factor 2 alpha kinase 2	Type II interferon signaling
NM_000639	FASLG	356	Fas ligand	Type II interferon signaling
NM_002053	GBP1	2633	guanylate binding protein 1	Type II interferon signaling
NM_000201	ICAM1	3383	intercellular adhesion molecule 1	Type II interferon signaling
NM_002176	IFNB1	3456	interferon beta 1	Type II interferon signaling

NM_000619	IFNG	3458	interferon gamma	Type II interferon signaling
NM_000576	IL1B	3553	interleukin 1 beta	Type II interferon signaling
NM_002198	IRF1	3659	interferon regulatory factor 1	Type II interferon signaling
NM_006084	IRF9	10379	interferon regulatory factor 9	Type II interferon signaling
NM_016816	OAS1	4938	2-5-oligoadenylate synthetase 1	Type II interferon signaling
NM_002800	PSMB9	5698	proteasome subunit beta 9	Type II interferon signaling
NM_007315	STAT1	6772	signal transducer and activator of transcription 1	Type II interferon signaling
NM_000593	TAP1	6890	transporter 1 ATP-binding cassette sub-family B MDR TAP	Type II interferon signaling
NM_013351	TBX21	30009	T-box 21	Type II interferon signaling
